# The impact of low-level lead toxicity on school performance among children in the Chicago Public Schools: a population-based retrospective cohort study

**DOI:** 10.1186/s12940-015-0008-9

**Published:** 2015-04-07

**Authors:** Anne Evens, Daniel Hryhorczuk, Bruce P Lanphear, Kristin M Rankin, Dan A Lewis, Linda Forst, Deborah Rosenberg

**Affiliations:** University of Illinois at Chicago, 2121 W. Taylor Street, Chicago, 60612 IL USA; University of Illinois at Chicago, Center for Global Health, College of Medicine, 1940 W. Taylor Street, Chicago, 60612 IL USA; Northwestern University, School of Education and Social Policy, 2120 Campus Drive, Evanston, 60208 IL USA; Child & Family Research Institute, BC Children’s Hospital and Faculty of Health Sciences, Simon Fraser University, Vancouver, V5Z 3E5 BC Canada

**Keywords:** Blood lead concentration, Health disparities, Lead poisoning, School performance, Standardized tests, Urban lead exposure

## Abstract

**Background:**

Environmental lead exposure poses a risk to educational performance, especially among poor, urban children. Previous studies found low-level lead exposure was a risk factor for diminished academic abilities, however, this study is distinct because of the very large sample size and because it controlled for very low birth weight and early preterm birth–two factors closely associated with lower academic performance. In this study we examined the association between lead concentration in whole blood (B-Pb) of Chicago Public School (CPS) children and their performance on the 3^rd^ grade Illinois Standard Achievement Tests (ISAT) reading and math scores.

**Methods:**

We examined 58,650 children born in Chicago between 1994 and 1998 who were tested for blood lead concentration between birth and 2006 and enrolled in the 3^rd^ grade at a CPS school between 2003 and 2006. We linked the Chicago birth registry, the Chicago Blood Lead Registry, and 3^rd^ grade ISAT scores to examine associations between B-Pb and school performance.

**Results:**

After adjusting for other predictors of school performance including poverty, race/ethnicity, gender, maternal education and very low birth weight or preterm-birth, we found that B-Pbs below 10 μg/dL were inversely associated with reading and math scores in 3^rd^ grade children. For a 5 μg/dL increase in B-Pb, the risk of failing increased by 32% for reading (RR = 1.32, 95%CI = 1.26, 1.39) and math (RR = 1.32, 95%CI = 1.26, 1.39). The effect of lead on reading was non-linear with steeper failure rates at lower B-Pbs. We estimated that 13% of reading failure and 14.8% of math failure can be attributed to exposure to blood lead concentrations of 5 to 9 vs. 0 to 4 μg/dL in Chicago school children.

**Conclusions:**

Early childhood lead exposure is associated with poorer achievement on standardized reading and math tests in the third grade, even at very low B-Pbs. Preventing lead exposure in early childhood is critical to improving school performance.

## Background

Lead is a known neurotoxin that has been associated with reduction in intellectual abilities, learning deficits, and neurobehavioral disorders in children. Epidemiologic studies of lead-exposed children indicate that the dose–response relationship between blood lead concentrations (B-Pbs) and IQ is non-linear, with proportionately greater loss of IQ points at lower B-Pbs [1,2].

A cross-sectional analysis of data from the Third National Health and Nutrition Examination Survey found that at B-Pbs below 5.0 μg/dl, a 1 μg/dl increase in B-Pbs was associated with a 0.7 point decrement in arithmetic and a 1-point decrement in reading scores on the Wide Range Achievement Test-Revised [3]. Miranda et al. linked blood lead surveillance date to educational testing data for 4^th^-grade students from North Carolina and found that B-Pbs below 10 μg/dl were associated with dose-related declines in end-of-grade testing scores in mathematics and reading [4]. Amato et al. compared educational proficiency in EOG testing on the Wisconsin Knowledge and Concepts Exam of 4^th^ grade children with B-Pbs < 5 μg/dL to children with B-Pbs between 10 – 19 μg/dL. Test score deficits between these two groups were equal to 22% of the interval between students categorization at the “proficient” level in Reading and 42% of the interval in Mathematics [5]. A study by Zahran et al. [6], which matched children’s blood lead concentrations and standardized test performance in 117 metropolitan New Orleans elementary schools, showed that for each unit change in median B-Pb concentration, there was an increase of 3.8 (95% CI, 2.2–5.5) in the percentage of children in the Unsatisfactory category. Zhang et al. [7] linked B-Pb surveillance data to Michigan Educational Assessment Program scores for children in grades 3, 5, and 8. The odds of scoring non proficient in mathematics, science and reading for children who had B-Pbs > 10 μg/dL were more than twice the odds for those whose B-Pbs were < 1 μg/dL [7].

We examined the effect of B-Pbs on performance on the Illinois Standard Achievement Test (ISAT) of 47,168 children enrolled in the 3^rd^ grade at a Chicago Public School between 2003 and 2006 by linking children’s B-Pbs, birth certificate records, and ISAT scores. This large sample size allowed us to examine the shape of the dose–response relationship between B-Pbs and failure on standardized tests in reading and writing of 3^rd^ grade children over a wide range of B-Pbs and explore disparities in the effects of lead on school performance by race (white, black, Hispanic). It also allowed us to calculate the percent of children in the Chicago School system who failed reading or math due to childhood lead exposure.

## Methods

This research is a collaborative effort of the Chicago Department of Public Health (CDPH), CPS and the University of Illinois at Chicago, School of Public Health (SPH). Existing data sets were linked to examine the association between early childhood B-Pbs, socio-demographic data, and school performance as measured by ISAT scores. All aspects of this study, including data linkage, analysis, and presentation of results, were conducted according to a protocol approved by the Institutional Review Board of the University of Illinois at Chicago.

### Inclusion criteria and study design

The study population is a cohort of children who were: born in the six-county area of metropolitan Chicago between 1994 and 1998; residents of Chicago during early childhood; had a B-Pb test reported to the CDPH between 1996 and 2006; and were enrolled in a CPS school with available ISAT scores between 2003 and 2006. There were 58,560 children who met these inclusion criteria, representing 39% of the 149,768 children enrolled in CPS in the third grade. We found no significant differences in the characteristics of children in the study compared with those enrolled in Chicago Public Schools, p < 0.0001 level, except that Hispanic children were under-represented in the study sample. This is likely because Hispanic children are much more likely to have immigrated to the US and do not have available birth registry data, thus making them ineligible for the study. We also excluded 1,136 children who were missing race/ethnicity. An additional 74 children had missing data for all of the outcome variables, resulting in a final sample size of 57,350 children. We used this full data set to investigate the shape of the dose–response relationship between B-Pbs and ISAT math and reading failure rates across the entire range of B-Pbs. Because the primary focus of this study, however, was to investigate the effects of low-level lead exposure on school performance, we restricted most analyses to the 47,168 children who had B-Pbs less than 10 μg /dL.

### Datasets

The dataset for this study was created by linking three administrative databases: the Chicago Blood Lead Surveillance Program, the Chicago Birth Registry, and CPS performance records.

### The blood lead surveillance dataset

Under the Illinois Lead Poisoning Prevention Act, laboratories are required to report the results of blood lead concentration tests for children 72 months or younger residing in Illinois to the Illinois Department of Public Health (IDPH). The IDPH shares the blood lead concentration tests with the CDPH Childhood Lead Poisoning Prevention Program (Chicago CLPPP). The Chicago CLPPP database of B-Pbs for children living in Chicago from 1996 to the present includes the following information: identification (child’s name, sex, date of birth, race, ethnicity, and, more rarely, SSN, Medicaid ID, or guardian’s name,), contact information (address, unit, zip code, and, more rarely, phone number) and test information (physician, laboratory, date of sample, analysis, age at the time of the test, type of test, and result). The Chicago CLPPP promoted venous testing and required follow-up testing for elevated capillary tests.

Blood lead venous samples were analyzed by the IDPH lab and by private laboratories using either ICPMS or atomic absorption spectroscopy. The Chicago Blood Lead Surveillance System (CBLS) collects and manages child-specific data with unique child, lab test result and address fields.

### The birth registry

We linked the B-Pb database with Chicago Vital Statistics to account for socio-demographic factors that might influence school performance. The CDPH Vital Statistics section manages birth certificate data that includes child’s date of birth, weight at birth, race/ethnicity, APGAR scores (a method of assessing the health of newborn children based on Appearance, Pulse, Grimace, Activity, Respiration), parental race/ethnicity and ages, maternal census tract, birthplace and maternal report of gestational smoking and alcohol intake, birth order, information on prenatal care, and clinical age of gestation.

### CPS school performance records

We used ISAT reading and math scores as a measure of school performance. ISAT data is collected as part of the Chicago Public Schools Student Information System (CPSIS), which tracks the academic progress of each CPS student. That data source includes the following data: identifying information (name, date of birth, sex, race, and ethnicity), contact information (address, unit, zip code, and phone number), Independent Education Plan (yes/no), Special Education (yes/no) and school performance information (reading, math, and composite standardized test scores).

### Data linking protocol

Linkage of these three datasets was performed by Chapin Hall, Center for Children at the University of Chicago, which maintains a data sharing and data analysis agreement with CDPH. Chapin Hall received B-Pb and birth registry datasets from CDPH. Chapin Hall matched these files to CPS data by first name, last name and date of birth using probabilistic matching methods. Exact matches were taken first, and then a SAS function called SPEDIS was used to conduct “fuzzy matching” that provides a numeric value for distance between matches and creates a list of probable matches. The probability of commonly found names was included in the matching protocol. The list of possible matches was then verified manually. Finally, we replaced all identifying information, including names and dates of birth which were used to link the files, with unique coded identifiers and deleted all individual identifiers.

### Exposure measures

Blood lead tests were used as a measure of childhood lead exposure. Most children in the study had only one recorded B-Pb in the matched blood lead surveillance dataset. For children with more than one blood lead test, the most recent venous blood lead concentration test was used (children with multiple B-Pbs accounted for less than 10% of the sample). Most children in the study (88%) had venous test results. For the purposes of this study, results included all B-Pbs (capillary and venous) as reported in the dataset. In secondary analyses, we examined the results using only venous B-Pbs and there were no appreciable differences in the results; therefore, all B-Pb tests were used.

### Outcome measures

The main outcome variable for this study was 3^rd^ grade ISAT scores, a measure of individual student achievement relative to the Illinois Learning Standard. The State of Illinois also uses the results to report student achievement to the public. The ISAT measures the achievement of students for reading and mathematics in grades three through eight and for science in grades four and seven. ISAT results used were for the years 2003 through 2006. The 2006 ISAT had a grade adjusted scale, allowing students from 3^rd^ through 8^th^ grade to be measured on the same underlying scale [8]. Additionally, the failure rate for reading and math tests was used as an outcome variable. Each year the reading and math test scores are divided into four categories: academic warning (failure), below standards, meets standards and exceeds standards. Children who get a score in the academic warning (failure) category will be retained and required to repeat the third grade unless they are retested and score higher.

### Covariates

The covariates used in this study (Table 1) were shown in other studies to be associated with academic achievement, including gender [9], poverty [10,11], race/ethnicity [12], maternal education [13], and the incidence of very low birth weight or preterm birth [14]. Gender was included as a potential confounder because previous literature has indicated that girls have higher performance than boys on standardized reading tests and perform slightly better on standardized math tests [9].

**Table 1:** **Demographic characteristics, mean blood lead concentrations, reading and math ISAT scores of Chicago cohort**

**Characteristic**	**n (%)**	**B-Pb (μg/dL) mean ± SD**	**Reading score ISAT mean ± SD**	**Math score ISAT mean ± SD**	**Reading failure %**	**Math failure %**
Overall	47,168 (100)	4.81 ± 2.22	154.9 ± 14.8	156.9 ± 14.2	14.6	14.5
Gender						
Male	23,746 (50)	4.85 ± 2.23	153.3 ± 14.8	156.6 ± 14.7	18.0	16.5
Female	23,413 (50)	4.77 ± 2.21	156.5 ± 14.6	157.2 ± 13.7	11.1	12.4
Race/Ethnicity						
Non-Hispanic black	30,020 (64)	5.25 ± 2.20	152.5 ± 14.2	152.5 ± 14.2	17.9	18.8
Hispanic	13,067 (28)	4.16 ± 2.03	157.6 ± 13.9	160.6 ± 13.4	9.6	7.5
Non-Hispanic white	4,081 (9)	3.72 ± 2.01	164.6 ± 15.9	167.2 ± 15.8	6.2	6.3
Mother’s education						
Some high school	19,233 (41)	5.09 ± 2.22	152.0 ± 13.9	154.3 ± 13.4	18.5	18.2
High school graduate	16,101 (34)	4.86 ± 2.23	154.4 ± 14.2	156.3 ± 13.8	14.4	14.5
Some college	8,554 (18)	4.49 ± 2.14	158.1 ± 14.6	159.6 ± 14.0	10.1	9.8
College graduate	2,274 (5)	3.88 ± 1.94	164.7 ± 15.4	166.5 ± 15.0	4.9	4.9
Post college	1,006 (2)	3.77 ± 1.92	169.2 ± 15.9	171.1 ± 15.6	3.6	2.8
Low-income						
Yes	41,289 (88)	4.93 ± 2.22	153.6 ± 14.1	155.6 ± 13.5	15.7	15.5
No	5,879 (12)	3.98 ± 2.02	164.0 ± 16.3	165.9 ± 15.9	7.0	7.0
Very low birthweight/early preterm						
Yes	1,955 (4)	5.12 ± 2.25	151.7 ± 14.3	152.8 ± 13.6	20.0	21.5
No	45,213 (96)	4.80 ± 2.21	155.1 ± 14.8	157.1 ± 14.2	14.4	14.2

Cohort includes **47,168 Children with B-Pbs <10 μg/dL** Summary statistics, including proportions, were calculated among those with non-missing data for that characteristic.

Reading and Math test scores are normally distributed as reported by the Illinois Standard Achievement Test (ISAT).

Note: Differences in mean blood lead concentrations, reading and math scores were found to be statistically significant (p < 0.0001) for all characteristics using standard *t*-test and ANOVA methods; Differences in proportions for reading and math failure were statistically significant (p < 0.0001) for all characteristics using chi-square tests.

Enrollment in the free or reduced-price lunch program was used as an indicator of family income to control for poverty and as a proxy for the income of the child’s family. Eligibility for the free or reduced-price lunch program, which is administered by the USDA, is based on income data submitted to CPS each year, and ranges from 1.3 to 1.85 times the federal poverty rate (approximately equivalent to an annual income of less than $45,000 for a family of four).

The race/ethnicity variable (data taken from the birth registry) includes Hispanic sub-populations. The distribution of race/ethnicity in the study sample is representative of the CPS student body, approximately two-thirds of which are African American, and one-quarter of which is Hispanic (Table 1). Analyses were stratified by race/ethnicity to test for effect modification.

The maternal education variable (data taken from the birth registry) measures the highest completed level of maternal education at the time of the child’s birth. The maternal education variable was coded into five categories (some high school, high school graduate, some college, college graduate and post college education), for compatibility with previous pediatric lead studies [4].

The incidence of very low birth weight or early preterm birth has previously been found to be the strongest birth outcome predictor of academic performance [14]. We created a dichotomous variable for the birth weight and preterm delivery based on data from the birth registry. We combined children who either had a very low birth weight (<=1.5 kg) or were very premature (gestational age ≤33 weeks) in one category for this analysis [14]. We adjusted for child’s age at the time of the blood lead test to account for age-related differences in blood lead concentrations. Children who had higher B-Pbs at 4 or 5 years of age usually had had higher B-Pbs at age 2 or 3 years of age. The typical peak exposure for children is 2 to 3 years of age, so a higher B-Pb at age 4 or 5 is usually indicative of a higher exposure over early childhood [1]. The mean age of the children at the time of the blood lead test is 45 months. Because a blood lead test is required for preschool, HeadStart, and kindergarten entrance in Chicago schools, children are typically tested as part of the enrollment process in an early education program. All of these blood lead tests occurred prior to the ISAT test, which typically occurs in the late spring of the child’s third grade year, between the child’s ninth and tenth birthday.

### Methods of analysis

Standard descriptive methods, multivariable linear regression, and log binomial regression were used to determine whether there is an association between childhood B-Pbs and school performance. Among the sample of children with B-Pb of <10 μg/dL (n = 47,168), we examined study variables using descriptive methods. Unadjusted analyses were conducted to determine the relationship between covariates and exposure and outcome variables using standard t-tests, analysis of variance, and chi-squares to test for differences in means or proportions between groups. We constructed multivariable linear regression models to determine the effect of B-Pbs < 10 μg/dL on reading and on math ISAT scores, both of which were normally distributed dependent variables. We first treated B-Pb as log-transformed B-Pb and secondly as a set of indicator variables representing each B-Pb, consistent with previous studies [1,4,8]. The models were compared through several test statistics (including the adjusted R^2^ and the root mean square error (RMSE)). The results of both the log-transformed B-PB analysis and the indicator variable version of B-Pb did not provide a better model fit than simply using untransformed B-Pb for lead exposure levels of < 10 μg/dL. Regardless of how we defined B-Pb, the estimated decline in ISAT scores was comparable. Therefore, the untransformed version was used for ease of interpretation. Next, we examined interaction terms to test for effect modification. The interaction terms between B-Pbs and race/ethnicity were found to be significant (p < 0.0001). Therefore, we stratified by race/ethnicity for all racial groups with sufficient sample sizes (non-Hispanic whites, non-Hispanic blacks, and Hispanics), and ran multivariable linear regression models that adjusted for all of the covariates mentioned previously.

School performance in reading and math was also measured using a dichotomous pass/fail outcome variable that was created by grouping those exceeding, meeting or falling below the standard as passing and those who failed the standard as failing. Multivariable log binomial regression models were used to produce relative risks and 95% confidence intervals for the relationship between B-Pb and failing vs. passing math and reading for the sample of children with B-Pbs <10 μg/dL.

To estimate the percentage of reading and math failure attributable to lead exposure of B-Pbs 5–9 vs. 0–4 μg/dL, we calculated the adjusted population attributable risk percent (PAR%), using the following equation:

$$ \mathrm{P}\mathrm{A}\mathrm{R}\ \%={\mathrm{p}}_{\mathrm{c}}*\left[\left(\mathrm{adjRR}\hbox{-} 1\right)/\mathrm{adjRR}\right], $$

where p_c_ is the prevalence of B-Pbs between 5–9 μg/dL among those who failed the reading and math test, respectively and adjRR is the adjusted relative risk from multivariable log binomial regression models that treated B-Pb as a dichotomous variable (5–9 vs. 0–4 μg/dL) and controlled for all covariates.

Finally, we repeated log binomial regression models for reading and math failure for the larger sample of children across the whole spectrum of B-Pbs, this time using the log transformed B-Pb, which provided a better fit to the data for the full range of B-Pbs, ranging from non-detectable levels to 120 μg/dL. Unadjusted and adjusted probabilities of failure for each B-Pb were estimated from a crude and multivariable model, respectively, and plotted to examine whether the slope of the relationship is different for B-Pb values < 10 μg/dL vs. ≥ 10 μg/dL. Adjusted probabilities were produced at the mean values of the covariates. An interaction term between the continuous B-Pb value and an indicator for values < 10 μg/dL vs. ≥ 10 μg/dL was added to this model to formally test the significance of a change in slope at 10 μg/dL. Although we used the full range of B-Pbs for models, we truncated the figures at 40 μg/dL due to high variance in unadjusted probabilities of failure for higher B-Pbs. SAS version 9.2 was used for all analyses [15].

## Results

The demographic characteristics including mean B-Pbs, reading and math scores of the 47,168 children with B-Pbs less than 10 μg/dL are shown (Table 1). B-Pbs were highest for non-Hispanic blacks, followed by Hispanics, and non-Hispanic whites. B-Pbs were also higher for males vs. females, low-income children, and children born at very low birth weights or early preterm. Mean B-Pbs decreased as the education level of the mother increased. 

Unadjusted mean reading and math scores, for all groups and stratified by race, decreased as B-Pbs increased from ≤ 2 to 9 μg/dL. The decline in performance on the reading and math tests was significant for all groups and for each of the race-stratified groups (p < 0.0001) (Table 2).

In multivariable linear regression analysis, we found that B-Pb was inversely associated with reading scores (p-value < 0.0001). The association between B-Pbs and reading scores shows that the decline in reading score per unit increase in blood lead concentration for all children was −0.60. Interaction terms between B-Pb and race/ethnicity were added to the model to test for effect modification by race/ethnicity and were found to be significant for blacks and Hispanics vs. whites (p = 0.01 and p = 0.006, respectively), but not significant for blacks compared to Hispanics (p = 0.39). The race specific associations were steepest for non-Hispanic white children (β = −0.76), followed by Hispanic children (β = −0.60), and then non-Hispanic black children (β = −0.57). The associated declines in reading scores are in the range of 5 to 7 points for a B-Pb increase of 9 μg/dL.

**Table 2:** **Model summaries for unadjusted and adjusted linear regression models for reading and math ISAT score (dependent variable)**

**Parameter**	**All races (n = 46,796)**			**Non-Hispanic white children (n = 4,064)**			**Non-Hispanic black children (n = 29,736)**			**Hispanic children (n = 12,996)**		
	**Estimate**	**SE**	**P**	**Estimate**	**SE**	**p**	**Estimate**	**SE**	**P**	**Estimate**	**SE**	**p**
Reading ISAT score												
*Unadjusted*												
B-Pb (μg/dL)	−1.28	0.03	<0.0001	−1.26	0.12	<0.0001	−0.92	0.04	<0.0001	−0.79	0.06	<0.0001
*Adjusted* ^a,b^												
B-Pb (μg/dL)	−0.60	0.03	<0.0001	−0.76	0.11	<0.0001	−0.57	0.04	<0.0001	−0.60	0.06	<0.0001
Math ISAT score												
*Unadjusted*												
B-Pb (μg/dL)	−1.22	0.03	<0.0001	−1.19	0.12	<0.0001	−0.76	0.03	<0.0001	−0.63	0.06	<0.0001
*Adjusted*												
B-Pb (μg/dL)	−0.50	0.03	<0.0001	−0.71	0.11	<0.0001	−0.44	0.03	<0.0001	−0.52	0.06	<0.0001

Model Summaries are for Children with B-Pbs lower than 10 μg/dL, Overall and Stratified by Race/Ethnicity^a^.

^a^For All Races, model includes blood lead concentration, gender, mother’s education, low-income, very low birth weight/early preterm, child’s age at time of B-Pb, ISAT vs. Iowa, and race (black vs. white and Hispanic vs. white).

^b^For individual races, model includes blood lead concentration, gender, mother’s education, low-income, very low birth weight/preterm, child’s age at time of B-Pb, ISAT vs. Iowa.

Multivariable linear regression models also showed that B-Pb was inversely associated with math score (Table 2). The decline in math scores per unit increase in blood lead for all children was −0.50, and the race specific associations were steepest for non-Hispanic white children (β = −0.71), followed by Hispanic children (β = −0.52), and then non-Hispanic black children (β = −0.44). Racial/ ethnic differences in slopes were significant for blacks vs. whites (p = 0.0002) and Hispanics vs. whites (p = 0.001), but not blacks vs. Hispanics (p = 0.56).

After adjusting for covariates, there was a 1.32 times (95% CI = 1.26, 1.39) increased risk of both reading and math failure associated with each 5 μg/dL increase in B-Pb (Table 3). These models also indicated significant interaction between race/ethnicity and B-Pb for non-Hispanic black children compared with non-Hispanic white children. The interaction for Hispanic versus white children was significant for reading, but not math failure. Among non-Hispanic white children, the relative risk of failing the 3^rd^ grade ISAT from a 5 μg/dL increase in B-Pb was 1.93 (95% C.I. 1.47, 2.54) for reading and 1.71 (95% C.I. 1.26, 2.30) for math. Among non-Hispanic black children, the relative risk of failing the 3^rd^ grade ISAT for each 5 μg/dL increase in B-Pb was 1.28 (95% C.I. 1.21, 1.35) for reading and 1.28 (95% C.I. 1.22, 1.35) for math. Among Hispanic children, the relative risk of failing the 3^rd^ grade ISAT from a B-Pb increase of 5 μg/dL was 1.47 (95% C.I. 1.29, 1.66) for reading and 1.51 (95% C.I. 1.31, 1.75) for math. Finally, we calculated the adjusted population attributable risk percent and found that 13.0% of reading failure and 14.8% of math failure can be attributed to exposure to blood lead concentrations of 5 to 9 μg/dL vs. 0–4 μg/dL. We further estimated that 25% of reading failure and 27% of math failure can be attributed to exposure to blood lead concentrations of 5 to 100 μg/dL vs. 0–4 μg/dL.

**Table 3:** **Log binomial regression models for the effect of blood lead concentration (B-Pb) on reading failure and math failure**

		**All children (n = 46,796)**		**NH white children (n = 4,064)**		**NH black children (n = 29,736)**		**Hispanic children (n = 12,996)**	
**Comparison**		**RR**	**95% CI**	**RR**	**95% CI**	**RR**	**95% CI**	**RR**	**95% CI**
Reading failure									
*Unadjusted* B-Pb (μg/dL)	1 μg/dL increase	1.12	1.11, 1.13	1.17	1.11, 1.23	1.08	1.07, 1.09	1.09	1.06, 1.12
	5 μg/dL increase	1.74	1.66, 1.83	2.19	1.67, 2.87	1.45	1.37, 1.53	1.54	1.36, 1.75
*Adjusted* ^a,b^ B-Pb (μg/dL)	1 μg/dL increase	1.06	1.05, 1.07	1.14	1.08, 1.20	1.05	1.04, 1.06	1.08	1.05, 1.11
	5 μg/dL increase	1.32	1.26, 1.39	1.93	1.47, 2.54	1.28	1.21, 1.35	1.47	1.29, 1.66
Math failure									
*Unadjusted* B-Pb (μg/dL)	1 μg/dL increase	1.13	1.12, 1.14	1.15	1.09, 1.22	1.08	1.07, 1.09	1.09	1.06, 1.13
	5 μg/dL increase	1.84	1.76, 1.93	2.03	1.51, 2.73	1.47	1.39, 1.55	1.58	1.37, 1.82
*Adjusted* B-Pb (μg/dL)	1 μg/dL increase	1.06	1.05, 1.07	1.11	1.05, 1.18	1.05	1.04, 1.06	1.09	1.06, 1.12
	5 μg/dL increase	1.32	1.26, 1.39	1.71	1.26, 2.30	1.28	1.22, 1.35	1.51	1.31, 1.75

Model Summaries are for Children with B-Pbs Lower than 10 μg/dL, Overall and Stratified by Race/Ethnicity.

^a^For All Races, model includes blood lead concentration (<10 μg/dL), gender, mother’s education, low-income, very low birth weight/preterm, child’s age at time of B-Pb, ISAT vs. Iowa, and race (black vs. white and Hispanic vs. white).

^b^For individual races, model includes blood lead concentration, gender, mother’s education, low-income, very low birth weight/preterm, child’s age at time of B-Pb, ISAT vs Iowa.

**Figure 1:**
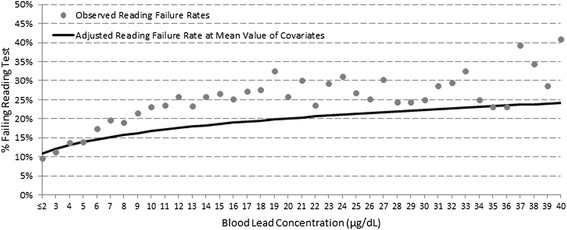
**Adjusted and observed reading failure rates by B-Pb.**

**Figure 2:**
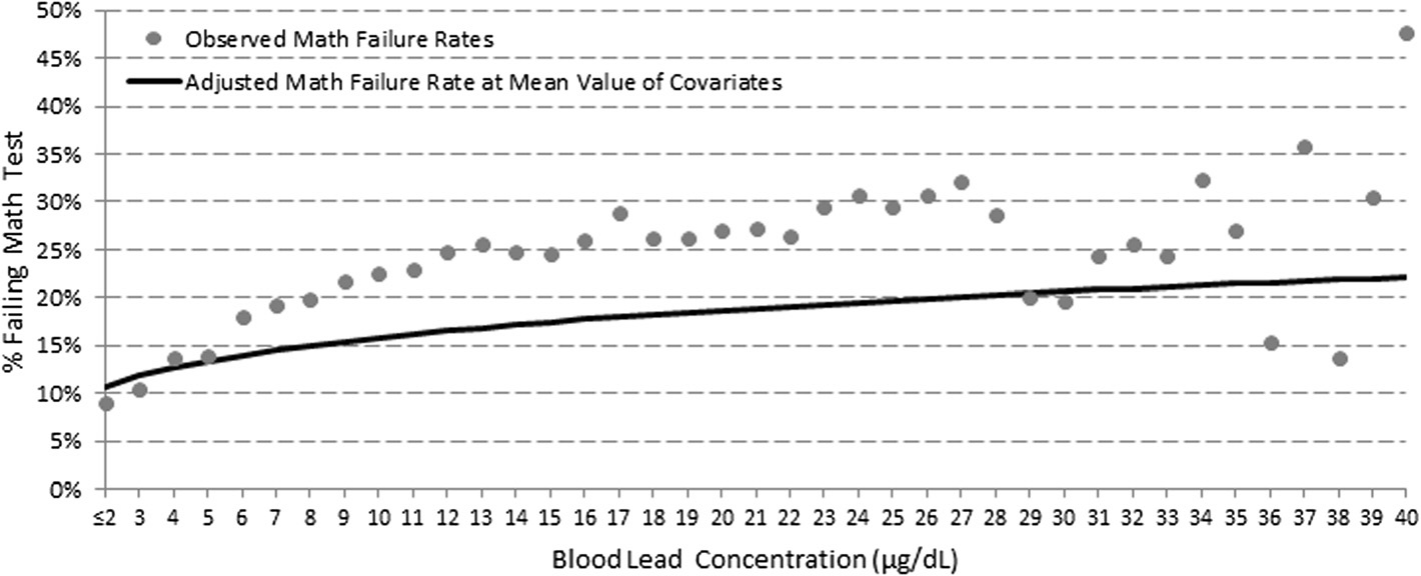
**Adjusted and observed math failure rates by B-Pb.**

To understand the implications of restricting our main analysis to children with B-Pbs <10, μg/dL, we ran models for the relationship between B-Pbs and failure rates for children with the whole spectrum of B-Pbs (including ≥10 μg/dL). Figures 1 and 2 show the observed unadjusted failure rates (displayed as circles) for reading and math failure, respectively. The solid line in each figure represents the adjusted (for all covariates mentioned above) slope for the relationship between B-Pb and failure rates, with adjusted probabilities of failure displayed at the mean value of the covariates. Adjustment leads to attenuation of the slope, but the positive relationships between B-Pb and reading and math failure remain significant. Adding an interaction term to the adjusted models to test for a change in slope at 10 μg/dL, revealed that the slope was significantly steeper for B-Pbs <10 vs. ≥10 μg/dL for reading (p = 0.01), but not math failure rates (data not shown).

## Discussion

We confirmed that early childhood B-Pbs are a major risk factor for poor academic achievement among 3^rd^ graders enrolled in CPS schools, even after controlling for gender, race/ethnicity, poverty, maternal education, very low birth weight and early preterm birth. Children who had higher B-Pbs scored significantly lower in the reading and math, and had significantly higher failure rates. These results clearly indicate that early childhood lead exposure has a negative impact on school performance, even at blood lead concentrations below 5 μg/dL. These results are consistent with the results of previous studies that observed dose-related declines in educational achievement measured by standardized tests with increasing B-Pb [4,7]. Our study further demonstrates that these declines occur at B-Pbs in the range of 2 – 9 μg/dL and that there are disparities in susceptibility by race/ethnicity that are not explained by gender, mother’s educational level, income or very low birth weight/pre-term birth. We also found that, for about one in six children, failure in reading or math is attributable to low-level lead toxicity.

We found that the impact of low-level lead toxicity varied by race and ethnicity. Consistent with other studies, we found the greatest disparities in lead exposure occurred among Non-Hispanic Black children, followed by Hispanics compared to Non-Hispanic Whites. We also found higher lead exposures among children who are lower income, born early preterm or very low birth weight, and/or born to mothers with less education. These factors are likely due to higher lead exposures resulting from residence in poorer quality housing and higher lead communities.

The steeper declines in achievement on standardized tests for Non-Hispanic whites with increasing B-Pbs compared to Non-Hispanic blacks and Hispanics was an unexpected finding. These differences in slopes remained after adjusting for gender, mother’s education, low income, and very low birth weight/pre-term birth. This paradox – disparities in exposure vs. disparities in effects – may be due to unmeasured environmental rather that biologic factors. Future studies that examine these disparities should attempt to include these additional confounders in their analyses.

The impact of low-level lead exposure on children’s school performance is substantial. Among all children, a 5 μg/dL increase in B-Pb within a B-Pb range of 2 – 9 μg/dL resulted in a 32% increase in the risk of failure in reading and math. We estimate that childhood lead exposure at levels ≥5 μg/dL accounted for as much as 25% of children failing in reading and math. Thus, while various risk factors are associated with school failure, lead exposure is a particularly important one. Moreover, it is entirely preventable.

We were primarily interested in studying the effects of low-level lead exposure on achievement on standardized tests. Our large sample size allowed us to investigate the shape of the dose–response relationship across the full range of B-Pbs. We observed a non-linear dose–response relationship: the increases in ISAT failure rates per 1 μg/dL increase of B-Pb were steeper at low B-Pbs (<10 μg/dL) than at higher B-Pbs. We tested the model for change in slope by including an interaction term (<10 μg/dL vs. ≥ 10 μg/dL) and found the change in slope to be significant for reading failure but not for math. Our observation of a non-linear dose–response relationship is consistent with the results of previous epidemiologic studies that demonstrated non-linear relationships between IQ and reading deficits and increasing B-Pbs [1,2].

There are several limitations to this study. First, although the sample population cohort was large and mostly representative of CPS students, it does not include immigrant children. Second, different labs reported different limits of detection for B-Pbs. Third, we were not able to adjust for parental IQ or HOME Inventory. Instead, we relied on surrogate measures, including maternal education and income. Finally, the use of free and reduced-price lunch as a proxy for economic status is potentially distorted because there is administrative pressure to maximize program eligibility, which could inaccurately inflate the number of families characterized as living in “poverty.”

## Conclusions

Our study confirms that small increases in B-Pbs, even at concentrations below 5 μg/dL, are associated with significant decrements in performance on standardized tests. Although B-Pbs have decreased over the past three decades across the whole of the United States, lead exposure remains a critical problem for large, urban, public school systems that are educating at-risk children. Preventing lead poisoning in early childhood is, therefore, an essential component of a strategy to improve the school success of lower income students.

## Abbreviations

APGAR: Appearance, pulse, grimace, activity, respiration
B-Pb: Blood lead concentration
CDPH: Chicago Department of Public Health
CPS: Chicago public schools system
CI: Confidence interval
ICPMS: Inductively coupled plasma mass spectrometry
IDPH: Illinois Department of Public Health
ISAT: Illinois standard achievement tests
PAR%: Population attributable risk percent
Root MSE: Root mean square error
